# Monitoring persistent pulmonary hypertension of the newborn using the arterial to end tidal carbon dioxide gradient

**DOI:** 10.1007/s10877-023-01105-2

**Published:** 2023-12-27

**Authors:** Emma E. Williams, Nadja Bednarczuk, Mahesh Nanjundappa, Anne Greenough, Theodore Dassios

**Affiliations:** 1 Women and Children’s Health, School of Life Course Sciences, Faculty of Life Sciences and Medicine, King’s College London, London, UK; 2 Neonatal Intensive Care Centre, King’s College Hospital, London, UK; 3 Neonatal Intensive Care Unit, University of Patras, Patras, Greece

**Keywords:** Carbon dioxide, Capnography, Mechanical ventilation, Right-to-left shunt

## Abstract

Persistent pulmonary hypertension of the newborn (PPHN) can be monitored theoretically by the difference of the partial pressure of arterial (PaCO_2_) to end-tidal CO_2_ (EtCO_2_). We aimed to test the hypothesis that the PaCO_2_–EtCO_2_ gradient in infants with PPHN would be higher compared to infants without PPHN. Prospective, observational study of term-born ventilated infants with echocardiographically-confirmed PPHN with right-to-left shunting and term-born control infants without respiratory disease. The PaCO_2_–EtCO_2_ gradient was calculated as the difference between the PaCO_2_ measured from indwelling arterial sample lines and EtCO_2_ measured by continuous Microstream sidestream capnography. Twenty infants (9 with PPHN and 11 controls) were studied with a median (IQR) gestational age of 39.5 (38.7–40.4) weeks, a birthweight of 3.56 (3.15–3.93) kg and a birthweight z-score of 0.03 (− 0.91 to 1.08). The PaCO_2_–EtCO_2_ gradient was larger in the infants with PPHN compared to those without PPHN after adjusting for differences in the mean airway pressure and fraction of inspired oxygen (adjusted p = 0.037). In the infants with PPHN the median PaCO_2_–EtCO_2_ gradient decreased from 10.7 mmHg during the acute illness to 3.3 mmHg pre-extubation. The median difference in the gradient was significantly higher in infants with PPHN (6.2 mmHg) compared to infants without PPHN (-3.2 mmHg, p = 0.022). The PaCO_2_–EtCO_2_ gradient was higher in infants with PPHN compared to term born infants without PPHN and decreased over the first week of life in infants with PPHN. The gradient might be utilised to monitor the evolution and resolution of PPHN.

## Introduction

**Fig. 1:**
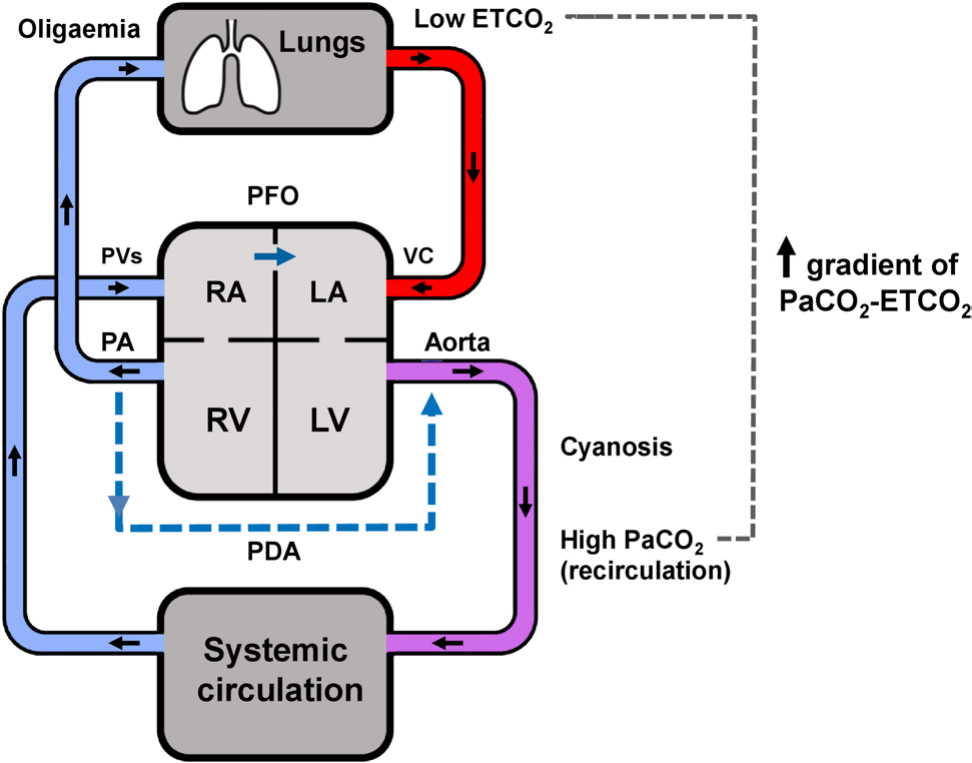
Schematic depiction of the effect of PPHN on the gradient of the arterial to end tidal CO_2_. Shunting increases the CO_2_ content of the arterial and capillary blood and lowers the pulmonary perfusion and thus the end-tidal CO_2_. *RA* right atrium, *RV* right ventricle, *LA* left atrium, *LV* left ventricle, *VC* vena cavae, *PA* pulmonary artery, *PVs* pulmonary veins, *PDA* patent ductus arteriosus, *PFO* patent foramen ovale, *PaCO*_2_ arterial partial pressure of CO_2_, ETCO_2_ end-tidal CO_2_

Persistent pulmonary hypertension of the newborn (PPHN) affects two in every 1,000 live births [1] and is characterised by increased pulmonary vascular resistance which could cause supra-systemic pulmonary pressures. This leads to shunting of deoxygenated blood from the pulmonary to the systemic circulation via the foramen ovale and the ductus arteriosus (extrapulmonary right-to-left shunting) [2]. PPHN is diagnosed and monitored by echocardiography which can detect the direction of shunting and estimate the pulmonary pressures [3]. The ensuing hypoxaemia and re-circulation of blood rich in carbon dioxide, lead to an increase in the partial pressure of carbon dioxide (PaCO_2_) in the arterial and capillary blood vessels (Fig. 1) [4]. Re-direction of blood from the pulmonary to the systemic circulation causes pulmonary oligaemia with some regions of the lung receiving less pulmonary blood volume, which is at mismatch with ventilation (alveolar dead space ventilation) [5]. The alveolar dead space can be calculated with a methodology which involves the offline construction and analysis of volumetric capnograms [6]. While these calculations can be methodologically challenging, the presence and magnitude of alveolar dead space can be clinically estimated by the difference of the arterial to the end-tidal carbon dioxide pressures (PaCO_2_–EtCO_2_ gradient) [7]. This gradient incorporates information not only on the alveolar dead space, but also on the anatomical and apparatus dead space [8], however these latter compartments would remain relatively constant for each individual infant during mechanical ventilation. Therefore, the fluctuation of this gradient may be able to be utilised to describe changes in right-to-left shunting in infants with PPHN.

We hypothesised that the arterial to end-tidal carbon dioxide gradient in term born infants with PPHN would be higher compared to term born infants without PPHN. Furthermore, the gradient would reduce in the PPHN infants as their condition improved. Our aim was to test these hypotheses.

## Methods

### Subjects

A prospective, observational study of ventilated infants at King’s College Hospital NHS Foundation Trust (KCH) Neonatal Intensive Care Unit was undertaken. Infants were recruited between January 2019 and March 2021. Ventilated term born infants (greater than 37 weeks of gestational age) with echocardiographically-confirmed PPHN with right-to-left shunting were included in the study. PPHN was diagnosed on the basis of echocardiographic parameters for the assessment of pulmonary artery pressure, pulmonary vascular resistance, right ventricular performance and shunts (3). The infants with PPHN underwent echocardiography for clinical confirmation of PPHN, which was suspected because of > 5% difference between pre and post-ductal transcutaneous saturation and/or an oxygen requirement of more than 50% which was not considered to be in accordance with the radiographic severity of the lung disease.

As a control group, term born infants who were ventilated for non-respiratory reasons with an oxygen requirement of less than 30% and without parenchymal lung disease were included. The included control infants were of similar gestational age to the infants with PPHN, as it has previously been described that the PaCO_2_–EtCO_2_ gradient is higher in infants of lower gestational age [8]. The control infants did not undergo echocardiography, as PPHN was not deemed clinically significant in the absence of an elevated oxygen requirement. All infants were ventilated with the SLE 6000 Neonatal Ventilator (Inspiration Healthcare, Crawley, UK) on volume targeted ventilation with a targeted volume of 5–6 ml/kg. The ventilator compensated for endotracheal tube leak up to 50%, according to the manufacturer. The study was approved by the London Camden and King’s Cross Research Ethics Committee and registered with the clinical governance department at KCH as part of a larger study determining the dead space and factors affecting the PaCO_2_–EtCO_2_ gradient in ventilated infants [6, 8].

### Assessment of the PaCO_2_–EtCO_2_ gradient

The PaCO_2_–EtCO_2_ gradient (in mmHg) was calculated concurrently to the echocardiographic assessment, as the difference between the partial pressure of arterial carbon dioxide measured from indwelling arterial sample lines and end-tidal carbon dioxide measured by continuous Microstream sidestream capnography. To assess the anticipated decrease of the PaCO_2_–EtCO_2_ gradient with the resolution of PPHN, the infants were assessed during their acute illness (when the echocargiography was performed) and also on the day prior to extubation—when the PPHN had clinically resolved and the infants were deemed by the clinical team to be ready for extubation, that is they had an oxygen requirement of less than 40%, a pH > 7.25 and a PaCO_2_ < 8.5 kPa, and their breathing rate was above the set ventilator rate.

### Clinical information

The following information was collected from the medical notes: gestational age (GA, weeks), birth weight (BW, kg), gender, and at the time of study the postnatal age (days), the fraction of inspired oxygen (FiO_2_), mean airway pressure (MAP, cmH_2_O) and targeted tidal volume (TTV, ml/kg). Standardised birth weight z-scores were calculated using the UK-WHO Royal College of Paediatrics and Child Health growth charts.

### Analysis

PPHN is uncommon and there is a paucity of published data on either the PaCO_2_–EtCO_2_ gradient or alveolar dead space in infants with PPHN. We therefore aimed to utilise a convenience sample of all infants with echocardiographically-confirmed PPHN admitted to the NICU during a two year period and a similar number of term ventilated infants without PPHN. Data were tested for normality and found to be non-normally distributed. Non-parametric tests were therefore utilised to determine if differences were statistically significant. Demographic and ventilatory parameters were compared between infants with PPHN and those without using the Mann Whitney U test. The difference in the gradient during the acute phase of the disease and the day prior to extubation was calculated in infants with PPHN and controls and compared between PPHN and controls using the Mann Whitney U test. Multivariable regression analysis was performed to determine if there was an independent relationship of PPHN with the arterial to end-tidal CO_2_ gradient after correcting for variables that significantly affected the gradient at the univariate level.

Statistical analysis was undertaken with SPSS version 28.0 (SPSS Inc., Chicago, IL, USA).

## Results

Twenty infants (13 male) were studied with a median (IQR) gestational age of 39.5 (38.7–40.4) weeks, a birthweight of 3.56 (3.15–3.93) kg and a birthweight z-score of 0.03 (− 0.91 to 1.08). Infants were first studied at a median of 4 (2–5) days. The controls were ventilated for poor perinatal adaptation, intestinal malrotation, spina bifida or neonatal stroke. The infants with PPHN all had an underlying diagnosis of meconium aspiration syndrome or confirmed or presumed neonatal sepsis.

The PaCO_2_–EtCO_2_ gradient during acute illness was larger in the infants with PPHN compared to those without (p < 0.001), and significantly related to both MAP (r = 0.62, p = 0.04) and FiO_2_ (r = 0.71, p < 0.001). The nine infants with PPHN were studied at a significantly earlier age than the controls (Table 1). The infants with PPHN had higher MAP (p < 0.001) and FiO_2_ (p < 0.001) requirements and a larger targeted tidal volume (p = 0.011). Linear regression analysis demonstrated that the PaCO_2_–EtCO_2_ gradient during acute illness was predicted by a diagnosis of PPHN [adjusted p = 0.037, 95% confidence intervals (CI) 0.7–18.0] after adjusting for differences in the MAP (p = 0.03) and FiO_2_ (p = 0.21). Tidal volume was not included in the model due to collinearity with FiO_2_ (r = 0.68, p = 0.03).

The PaCO_2_–EtCO_2_ gradient was assessed again before extubation. The infants with PPHN were assessed after a median (IQR) duration of ventilation of 9 (4–13) days. The median (IQR) PaCO_2_ in infants with PPHN before extubation was 41.5 (36.0–45.0) mmHg and the EtCO_2_ was 36.8 (28.0–40.8) mmHg. In infants with PPHN the median (IQR) PaCO_2_–EtCO_2_ gradient decreased from 10.7 (8.8–14.8) mmHg during the acute illness to 3.3 (2.2–3.7) mmHg pre-extubation, the median (IQR) difference in the gradient was thus 6.2 (5.1–9.4) mmHg. The control infants were extubated after a median (IQR) of 6 (4–8) days. The median (IQR) PaCO_2_ in the control infants before extubation was 42.3 (38.2–51.0) mmHg and the EtCO_2_ was 33.0 (30.8–36.0) mmHg. In the controls infants the median (IQR) PaCO_2_–EtCO_2_ gradient was 3.7 (1.7–4.8) mmHg during the acute illness and 7.2 (3.8–8.7) mmHg before extubation, the median difference in the gradient was thus − 3.2 mmHg. The median difference in the gradient was significantly higher in infants with PPHN compared to infants without PPHN (p = 0.022).

The median fraction of inspired oxygen (FiO_2_) was 0.21 in the controls during the acute illness and 0.25 pre-extubation. In infants with PPHN the median FiO_2_ was 0.75 during the acute illness and 0.37 pre-extubation. The mean airway pressure was similar during the acute illness and pre-extubation in the control infants (7.6 versus 7.9 cmH_2_O) and infants with PPHN (11.2 versus 10.8 cmH_2_O).

## Discussion

We have demonstrated that the gradient of the arterial to end tidal carbon dioxide was higher in infants with PPHN compared to term born infants of a similar gestational age without PPHN. Furthermore the gradient decreased from acute illness to the point before extubation in infants with PPHN.

In this study we report a PaCO_2_–EtCO_2_ gradient of 3.7 mmHg in the control infants when studied acutely which is not dissimilar to the median value of 3.3 mmHg in those with PPHN at the point of pre-extubation. Those values are in agreement with a previous study of term infants by our group [6], and the commonly described reference range of the PaCO_2_–EtCO_2_ gradient which has been reported to be between 2 and 5 mmHg [9, 10], even in health. The larger early gradient seen in our infants with a diagnosis of PPHN (10.7 mmHg) may therefore be attributable to right-to-left cardiac shunting, as the contribution of the anatomical and apparatus dead space would not change significantly for the same individual. The fact that the gradient decreases in PPHN but does not stay constant or reduce in the controls also points to the observation that the gradient is predominantly due to right-to-left to shunting although an element of improvement of the underlying pulmonary disease in the PPHN infants might also have contributed to this change. The infants with PPHN had higher inspired oxygen concentrations compared to the controls, despite being matched for basic demographics at birth. Oxygen is a pulmonary vasodilator and often utilised as a mainstay of treatment in PPHN [11], it is expected then that those with PPHN will have higher FiO_2_ levels regardless of pulmonary disease.

Our study has some potential clinical applicability. If our findings are replicated in the future in larger, independent cohorts, the PaCO_2_–EtCO_2_ gradient could be utilised in infants diagnosed with PPHN to monitor the evolution of right-to-left shunting and the response to therapy and resolution of PPHN. There is scope that this index could also be utilised clinically in conjunction with the oxygenation index. Indeed, resolution of PPHN would manifest both with an improvement in oxygenation and a decrease in the alveolar dead space (PaCO_2_–EtCO_2_ gradient). Additionally, one benefit of the PaCO_2_–EtCO_2_ gradient measurements is that they do not need indwelling arterial access as previous studies have reported that capillary PaCO_2_ values accurately reflect those measured arterially [12].

Our study has strengths and some limitations. The infants had echocardiographically confirmed PPHN with right-to-left shunting. We utilised a validated Microstream sidestream Capnograph [13] to measure the end-tidal CO_2_. One limitation of this study is the relatively small number of infants with PPHN. Given that the disease is rare, despite the study taking place in a tertiary neonatal unit, there were only nine infants with PPHN over a two year period. We compared, however, their results with control infants and demonstrated a significant difference in the PaCO_2_–EtCO_2_ gradient between the groups. We also note that in the control infants we did not exclude PPHN with echocardiography. The low oxygen requirement in these infants, however, would have ensured that no severe hypoxaemic PPHN was overlooked.

In conclusion, we have demonstrated that the arterial to the end-tidal CO_2_ gradient is larger in infants with PPHN compared to term infants without PPHN. We suggest this gradient might be utilised to monitor the evolution and resolution of PPHN.

**Table 1:** Characteristics of the infants according to a diagnosis of PPHN or no PPHN. Data are presented as median (IQR) or N (%)

	No PPHN (N = 11)	PPHN (N = 9)	p value
Gestational age (weeks)	39.1 (37.6–40.0)	40.3 (39.3–40.8)	0.090
Birth weight (kg)	3.34 (2.86–3.83)	3.60 (3.29–4.20)	0.222
Birthweight z-score	− 0.18 (− 1.57 to 0.59)	0.10 (− 0.44 to 1.45)	0.158
Gender (male)	7 (63.6)	6 (66.7)	1.000
Postnatal day (day)^a^	5 (4–5)	2 (1–7)	0.028
PaCO_2_ (mmHg)^a^	37.7 (34.5–38.9)	47.2 (43.4–48.3)	0.005
EtCO_2_ (mmHg)^a^	34.1 (30.4–36.8)	33.4 (29.3–37.1)	0.897
PaCO_2_–EtCO_2_ gradient (mmHg)^a^	3.7 (0.9–4.8)	10.7 (8.3–16.8)	< 0.001
Mean airway pressure (cmH_2_O)^a^	7.6 (6.4–8.2)	11.2 (9.7–12.3)	< 0.001
Fraction of inspired oxygen^a^	0.23 (0.21–0.30)	0.75 (0.55–0.79)	< 0.001
Tidal volume (ml/kg)^a^	5.0 (5.0–5.5)	6.0 (5.8–7.0)	0.011

^a^During acute illness

## Data Availability

Data is available upon reasonable request.
